# A survey of lifestyle factors in dystonia

**DOI:** 10.1002/brb3.1871

**Published:** 2020-10-06

**Authors:** Jacob Söderlund, Dag Nyholm

**Affiliations:** ^1^ Department of Neuroscience, Neurology Uppsala University Uppsala Sweden

**Keywords:** dystonia, lifestyle, physical activity, smoking

## Abstract

**Background:**

Knowledge about what causes dystonia is highly incomplete, especially about the impact of nongenetic factors.

**Aims of the Study:**

This cross‐sectional survey‐based explorative study examined different nongenetic factors in patients with dystonia.

**Methods:**

Information from both medical records and a questionnaire was collected. In total, 220 patients with dystonia were identified, of which 51 participated in the survey.

**Results:**

Women had a higher prevalence for cervical dystonia than men. Smoking was approximately twice as common in our studied population compared to the general Swedish population. Significantly more men than women met the criteria for low level of physical activity, yet the proportion of missing data was high in this category.

**Conclusions:**

Conclusions on causality cannot be drawn in this preliminary study, further research is encouraged regarding the link between smoking and dystonia.

## Graphical abstract

Knowledge about what causes dystonia is highly incomplete, especially about the impact of non‐genetic factors. This cross‐sectional survey‐based study examined different non‐genetic factors in patients with dystonia. Smoking was approximately twice as common in our studied population compared to the general Swedish population.

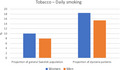

## 1 INTRODUCTION

“Dystonia is a movement disorder characterized by sustained or intermittent muscle contractions causing abnormal, often repetitive, movements, postures, or both. Dystonic movements are typically patterned, twisting, and may be tremulous. Dystonia is often initiated or worsened by voluntary action associated with overflow muscle activation.” (Albanese et al., 2013).

Dystonia was first described in 1911, but still today the definition is being updated and debated (Albanese et al., 2013; De Pablo‐Fernandez & Warner, 2017). The latest updated definition, quoted above, was presented in 2013. In addition to motor symptoms, increasing evidence indicates that nonmotor symptoms are troublesome for patients (Stamelou et al., 2012), leading to reduced quality of life (Müller et al., 2002; Timmers et al., 2017). Nonmotor symptoms can be pain, impaired sensory functions, neuropsychiatric disorders, sleep disturbances, and cognitive disturbances (Kuyper et al., 2011; Stamelou et al., 2012).

There is a reported connection between dystonia and psychiatric disorders, such as generalized anxiety disorder, obsessive–compulsive disorder, alcohol dependence, and depression. Whether these symptoms are effects of dystonia or side effects of treatment remains unclear.

A coherent understanding about the prevalence and risk factors for dystonia is still missing. Studies on prevalence of dystonia present varying numbers, ranging from 5 to 40 per 100,000 (Government of Canada and Neurological Health Charities Canada, 2014; Steeves et al., 2012) A systematic review by Krewski et al. (2017) found possible susceptibility to cervical dystonia among females. For blepharospasm, they proposed that there is no significant gender difference. Higher, rather than lower, consumption of coffee and alcohol has shown to be protective for onset of specific forms of dystonia, blepharospasm, and myoclonal dystonia, respectively, although the protective effect of alcohol is debated (Krewski et al., 2017).

An Australian case–control study with 184 patients and 1,048 controls showed significant association between idiopathic isolated dystonia (IID) and five different nongenetic factors: tremor, cigarette smoking, anxiety, depression, and head trauma with loss of consciousness (Newman et al., 2014). However, there is an ongoing debate if anxiety is just a confounding factor (Krewski et al., 2017) or a risk factor in itself. Except for this, data on lifestyles associated with dystonia are sparse.

We hypothesize some lifestyle factors, such as smoking, the use of snuff, alcohol consumption, physical activity, or dietary patterns in patients with dystonia, might be different from the general Swedish population. The purpose of this study was to learn more about nongenetic factors and their correlation with dystonia.

## 2 MATERIALS AND METHODS

Based on ICD‐10 codes G24.1–G24.9, 220 patients with dystonia were identified, during 2017, in the hospital records. Information about these patients’ age, sex, specific dystonia diagnosis, year of diagnosis, and any other neurological diseases were retrieved from electronic or scanned medical records.

Before their next appointment, information about the study, and the questionnaire to fill out was sent to the patients, by postal mail. During the study period, a total number of 170 patients had an appointment for regular intramuscular botulinum toxin injection, all were considered to respond to the treatment. Of these patients, 55 patients (32%) answered the questionnaire. Patients who were 17 years or younger, had acquired dystonia, or dementia were excluded (*n* = 4) from the survey, leaving 51 for further analysis. None of these had generalized or hereditary dystonia.

This project was approved by the Regional Ethical Review Board in Uppsala, and written informed consent was obtained from all participants.

The questionnaire contained two parts. The first part consisted of questions related to diagnosis, education, and caffeine consumption. The second part was a standardized questionnaire, with questions about lifestyle, more specifically about exercise, the use of tobacco and alcohol, and dietary habits.

In 2011, The National Board for Health and Welfare (NBHW) in Sweden presented guidelines, regarding methods for preventing disease (National Board for Health & Welfare in Sweden, 2011). The guidelines define lifestyle habits leading to an increased risk for various diseases according to Table 1.

**Table 1:** Guidelines from The National Board for Health and Welfare in Sweden, regarding methods for preventing disease (National Board for Health & Welfare in Sweden, 2011)

Smoking—Smoking daily, regardless of number of cigarettes.
Snuff—Daily use of snuff, regardless of amount of snuff.
Hazardous drinking (alcohol)—more than 9/14 (women/men) standardized units of alcohol per week, or 4/5 (women/men) or more units at one occasion, one or more times per month.
Low level of physical activity—One who does not meet the requirements of 150 min per week of moderate activity (e.g. walking, bicycling and gardening) or 75 min per week of intense activity (e.g. running and other sports).
Unhealthy eating habits—0–4 points out of 12 points, calculated from 4 questions about weekly intake of vegetables, fruit, fish and pastry/candy/crisps. Each answer renders 0–3 points depending on how frequent the intake is.

The second part of the questionnaire included 11 questions, which are based on the same guidelines and recommended by the NBHW, for how healthcare personnel can and should investigate patients’ lifestyle.

Data from all questionnaires were processed in Microsoft Office Excel 2013 and IBM SPSS Statistics 25. When comparing women with dystonia to men with dystonia, the p‐value was calculated using chi‐square test. Level of significance was set to 0.05.

## 3 RESULTS

Mean age of all 220 patients was 63 years (*SD* ± 15 years). Time since dystonia diagnosis ranged from 0 to 61 years, with median (interquartile range) of 12.6 years (3.9–21.5 years). Of these patients, 154 (70%) were women, 145 (66%) had cervical dystonia, and 176 (80%) had no other recorded disease, except for dystonia. Significantly more women had cervical dystonia, but no gender differences were seen in the smaller groups of blepharospasm, oromandibular dystonia, hereditary dystonia, other specified dystonia, or unspecified dystonia.

In the group (*n* = 169) that did not complete the survey, 105 (62%) had cervical dystonia, with the remaining dystonia types as follows: other specified dystonia (*n* = 20), blepharospasm (*n* = 17), unspecified dystonia (*n* = 17), orofacial dystonia (*n* = 9), and hereditary dystonia (*n* = 1).

A total number of 51 patients were included in the survey, 75% were women (Table 2). Mean age for women was 68 years (*SD* ± 10.4 years) and for men 67 years (*SD* ± 12.7 years). Seventy‐five percent of the patients had cervical dystonia, the remaining had blepharospasm, oromandibular dystonia, or other specified dystonia. Only two patients had parkinsonism, one with orofacial dystonia and one with hemifacial spasm.

**Table 2:** Demographics of survey responders (*N* = 51)

Diagnosis	*n*	Sex (F/M)	Mean age (years)	Mean disease duration (years)
Cervical dystonia	38	29/9	66.6	16.0
Idiopathic orofacial dystonia	3	3/0	72.0	13.9
Blepharospasm	4	4/0	75.3	14.7
Other specified dystonia	2	0/2	62.5	8.5
Hemifacial spasm	4	2/2	70.8	5.8
TOTAL	51	38/13	67.7	14.7

Patients’ answers to lifestyle questions are presented in Table 3. There was a gender difference in terms of low level of physical activity, where men were significantly more inactive than women. The seven men who were most inactive had cervical dystonia (*n* = 4), hemifacial spasm (*n* = 2), and other specified dystonia (*n* = 1), thus with similar distribution of dystonia subtypes compared with the whole sample. For visual comparison, statistics for the same questions from the general population is presented, collected by the Public Health Agency of Sweden (PHAS) and the NBHW. Daily tobacco smoking was twice as common in our dystonia patients as in the general Swedish population. Statistical analysis was not performed due to the small number of dystonia patients. Furthermore, only 31.6% of the women in our study had never been smokers.

**Table 3:** Lifestyle in dystonia patients

	Total, *N* = 51 Women, *n* = 38 Men, *n* = 13	Missing answers	Dystonia		General Swedish population %
			Patients	%	
Tobacco – Daily smoking	Total	0	9	17.6	8.8
	Women	0	7	18.4	10
	Men	0	2	15.4	8
Tobacco – Daily use of snuff	Total	3	4	8.3	11
	Women	1	2	5.4	4
	Men	2	2	18.2	18
Alcohol – Hazardous drinking	Total	4	6	12.8	17^a^
	Women	2	3	8.3	12^a^
	Men	2	3	27.3	21^a^
Low level of physical activity^b^	Total	7	13	34.2	35
	Women	6	**6**	**18.8**	36
	Men	1	**7**	**53.8**	35
Unhealthy dietary habits	Total	0	5	9.8	20
	Women	0	4	10.5	Unknown
	Men	0	1	7.7	Unknown

Note

Bold characters visualize a significant difference (*p* < .05) between women and men with dystonia.

^a^ The numbers are specific for Uppsala county.

^b^ Patients who fulfilled none of the two physical activity criteria, 75 and 150 min.

## 4 DISCUSSION

Knowledge about nongenetic factors and their correlation to dystonia is very limited. This study aimed at learning more, through a lifestyle questionnaire sent out to 170 patients with dystonia. It should be considered as a preliminary study looking for any correlations with lifestyle factors to suggest further studies on causality or possible risk factors. The response rate was rather low, 32%, which is an interesting finding in itself. Our experience with other movement disorders, such as Parkinson's disease (PD), is quite different. PD patients generally have much higher response rates in similar studies, and it is also a group that is well‐known for nonuse of tobacco and caffeine (Marras et al., 2019).

In the present study, age did not differ between women and men with dystonia, which is consistent with previous studies. However, significantly more women were diagnosed with dystonia, compared to men. This has been described in previous studies of all types of dystonia (Pekmezović et al., 2003) and cervical dystonia (Marras et al., 2007) Among patients with cervical dystonia, significantly more were female than male. These findings are consistent with those of Krewski et al. (2017) but opposite to findings from Steeves et al. (2012).

When comparing answers from patients’ response to the questions about lifestyle, the only significant finding of differences between women and men with dystonia is for physical activity. Significantly more men than women (53.8%–18.8%) met the criteria for low level of physical activity. But, a noticeable number of answers were missing for women compared to men (6 vs. 1). If all missing answers were to be considered as “Low level of physical activity,” the significant difference would vanish. Physiotherapy is an important part of the management of dystonia (De Pauw et al., 2014) and encouraging physical activity is important in clinical practice, because it is strongly associated with quality of life (Zetterberg et al., 2009).

Newman et al. (2014) have reported an association between idiopathic isolated dystonia and cigarette smoking, and a case report demonstrated that smoking worsened the cranial dystonia in one patient (Prashantha & Pal, 2009). In our population, we conclude that the prevalence of smoking is approximately twice that of general Swedish population. Furthermore, only 31.6% of the women in our study had never been smokers. This is a rather low number compared to statistic from PHAS, which shows that the corresponding number for women in Uppsala county is 66%. This is an interesting finding, but it is important to clarify that no calculations on level of significance have been made between patients with dystonia and general population.

The main limitations with the present explorative study are the small number of patients and the lack of a specific control group. The patients who participated in this study, by filling out the questionnaire, might not be representative for the entire population of people with dystonia in Uppsala. Several dystonia diagnoses were included. On one hand, this gives strength to general conclusions about dystonia. But on the other hand, it makes conclusions about specific dystonia diagnoses more uncertain. The patients who responded to the survey had five different dystonias, with cervical dystonia as the predominant type. All patients were in the regular botulinum toxin program, and none had generalized or hereditary dystonia.

To conclude, we have shown that significantly more women than men have dystonia at our hospital. The results strengthen previous findings that cervical dystonia predominantly affects women. Regarding lifestyle, we found significantly more men who had a low level of physical activity, compared to women, yet these had missing response to a higher extent. Interestingly, present or previous smoking was much more common in the dystonia patients than in the general population. Conclusions on causality cannot be drawn in this preliminary study, further research is encouraged.

Finally, dystonia causes great social stigmata for many patients. This implicates that more knowledge and better understanding of dystonia must be obtained.

## CONFLICT OF INTEREST

The authors report no disclosures/conflict of interest and no external funding.

## AUTHOR CONTRIBUTION

Both authors have made substantial contributions to conception and design, acquisition of data, analysis and interpretation of data; been involved in drafting the manuscript (JS) or revising it critically for important intellectual content (DN); and given final approval of the version to be published. Both authors have participated sufficiently in the work to take public responsibility for appropriate portions of the content; and agreed to be accountable for all aspects of the work in ensuring that questions related to the accuracy or integrity of any part of the work are appropriately investigated and resolved.

### Peer Review

The peer review history for this article is available at https://publons.com/publon/10.1002/brb3.1871.

## Data Availability

The data that support the findings of this study are available on request from the corresponding author. The data are not publicly available due to privacy or ethical restrictions.
